# Efficient purification and concentration of viruses from a large body of high turbidity seawater

**DOI:** 10.1016/j.mex.2014.09.001

**Published:** 2014-09-16

**Authors:** Guowei Sun, Jinzhou Xiao, Hongming Wang, Chaowen Gong, Yingjie Pan, Shuling Yan, Yongjie Wang

**Affiliations:** a Laboratory of Quality and Safety Risk Assessment for Aquatic Products on Storage & Preservation (Shanghai), Ministry of Agriculture, China; b College of Food Science and Technology, Shanghai Ocean University, Shanghai, China; c Institute of Biochemistry and Molecular Cell Biology, University of Göttingen, Germany

**Keywords:** Concentration of marine viruses, Marine viruses, Concentration, Tangential flow filtration, Ultrafiltration, High turbidity seawater

## Abstract

Marine viruses are the most abundant entities in the ocean and play crucial roles in the marine ecological system. However, understanding of viral diversity on large scale depends on efficient and reliable viral purification and concentration techniques. Here, we report on developing an efficient method to purify and concentrate viruses from large body of high turbidity seawater. The developed method characterizes with high viral recovery efficiency, high concentration factor, high viral particle densities and high-throughput, and is reliable for viral concentration from high turbidity seawater. Recovered viral particles were used directly for subsequent analysis by epifluorescence microscopy, transmission electron microscopy and metagenomic sequencing. Three points are essential for this method:

• The sampled seawater (>150 L) was initially divided into two parts, water fraction and settled matter fraction, after natural sedimentation.
• Both viruses in the water fraction concentrated by tangential flow filtration (TFF) and viruses isolated from the settled matter fraction were considered as the whole viral community in high turbidity seawater.
• The viral concentrates were re-concentrated by using centrifugal filter device in order to obtain high density of viral particles.

## Graphical abstract

**Figure:**
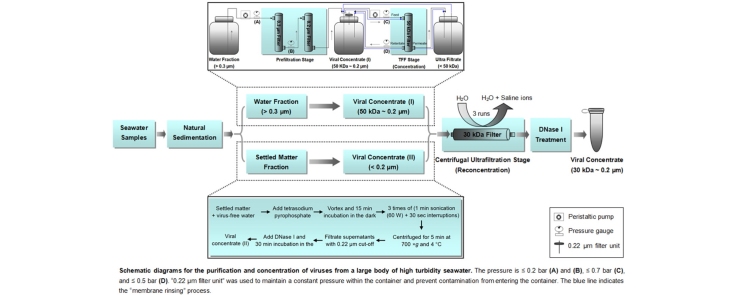

## Method details

### Sample preparation

More than 300 L of subsurface water (2 m depth), characterized by highly suspended matter contents (approximately 3.4 g L^−1^ (wet weight)), was sampled from Yangshan Deep-Water Port, South East Shanghai, China. Among them, 500 mL was fixed *in situ* by adding 0.02 μm filtered formalin (37–40% (w/v) formaldehyde solution) (Sangon, Shanghai, China) to 2% (v/v) final concentration for enumeration of viruses in the original water sample. The collected water samples were kept on ice and delivered to the laboratory as quickly as possible after finishing sampling.

### Enumeration of viruses in original seawater sample

**Fig. 1:**
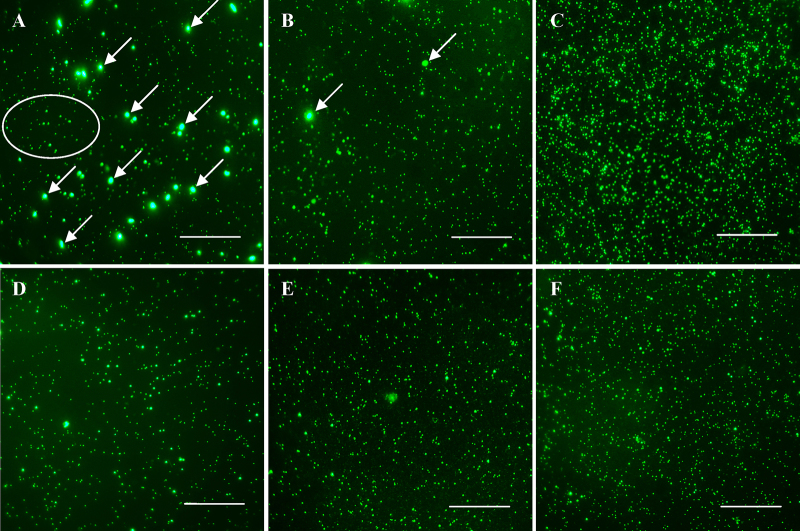
Epifluorescence-microscope image of each step's samples filtered onto a Whatman 0.02 μm Anodisc filter and stained with SYBR Green I. (A) Original seawater; (B) sample “A” filtered by 0.3 μm and 0.2 μm filters; (C) sample “B” concentrated by 50 kDa TFF ultrafilter and diluted 100 times; (D) eluant samples of membrane rinsing process diluted 10 times; (E) viral concentrate from settled matter diluted 100 times; (F) samples “C + D + E” reconcentrated by 30 kDa Centricon Plus-70 ultrafilter (Millipore) and diluted 15,000 times. The arrows indicate prokaryotes and the ellipse indicates >30 KDa virus-like particles. Scale bar = 20 μm. Note: a small number of prokaryotes appear in (B), and their possible origins are from the instruments and environment. They were removed after TFF by using the 0.22 μm cut-off filter unit.

In order to assess the recovery efficiency of the method established in this study for purification and concentration of marine viruses from high turbidity seawater, viral particles in the original seawater samples (Table 1 and Fig. 1A) were initially determined by epifluorescence microscopy after SYBR Green I staining according to the procedure described in [1,2]. All enumeration of viral particles in this study was performed by using the identical protocols.

### Concentration of viruses from the high turbidity seawater

The procedures for purification and concentration of virus particles from water samples are outlined in Graphical Abstract. The major procedures are as followed:

1. *Sedimentation by natural gravity*. 150 L of seawater samples were maintained in the dark for 12 h at 4 °C. After sedimentation, the samples were divided into two parts: water and settled matter fractions. The water fraction (approximately 150 L) was subsequently subjected to viral concentration, and the settled matter (approximately 509.8 g) was stored at −80 °C before viruses were isolated and concentrated.
2. *Removal of all particles or cells larger than 0.2 μm*. The water fraction (approximately 150 L) was successively filtered through 0.3 and 0.2 μm pore-sized filters in a stainless steel filter holder, a high performance and throughput filtration system (Millipore, MA, USA) equipped with the reusable cartridge filter with a large surface area, under a low entry pressure (<0.2 bar) driven by a peristaltic pump (Millipore, MA, USA). Afterwards, the “viral fraction” seawater (the filtrate, approximately 150 L) was obtained. The number of viral particles in the filtrate (Table 1 and Fig. 1B) was determined by epifluorescence microscopy after SYBR Green I staining.
3. *TFF concentration*. The “viral fraction” seawater (approximately 150 L) was subsequently concentrated by using a large-scale TFF system with 50 kDa cut-off tangential flow filter (Millipore, MA, USA) (see Graphical Abstract). The intake pressure driven by the peristaltic pump was below 10 p.s.i. (approximately 0.7 bar) to protect viruses from being destroyed, resulting in loss of virus yield [3]. When the volume of “viral fraction” seawater was less than 1 L, the viral concentrate was transferred into a sterilized container (*i.e.* viral concentrate (I), see Graphical Abstract). The number of viral particles in ‘viral concentrate (I)’ (Table 1 and Fig. 1C) was determined by epifluorescence microscopy after SYBR Green I staining.
4. *Membrane rinsing.* 20 L of TFF permeate (virus-free seawater) (see Graphical Abstract) was used to rinse the tangential flow filter until the water volume was less than 1 L. This part of the eluant, containing viruses trapped on the filter membrane and connection hoses during the first TFF, was also incorporated into the viral concentrate (I) (we call this step “membrane rinsing”). Precautions and operating techniques in TFF are described in detail in [3]. The number of viral particles in the eluant samples (Table 1 and Fig. 1D) was determined by epifluorescence microscopy after SYBR Green I staining.
5. *Isolation of viruses from settled matter*. Approximately 4 g of frozen settled matter was transferred to a 50 mL sterile centrifuge tube containing 5 mL virus-free seawater (sterile 50 kDa TFF permeate). Then 0.7 mL of tetrasodium pyrophosphate solution (10 mM final concentration) was added, and the slurry was shaken for 1 min with a ShakeMaster (Eppendorf, Hamburg, Germany) at maximum speed. After incubation for 15 min in the dark, the slurry was sonicated for 3 min (3× 1 min) using a 60 W sonication probe (Xinzhi, Ningbo, China). To prevent overheating, sonication treatment was performed at intervals of 1 min with 30 s interruptions in an ice bath. The sonicated slurry was centrifuged for 5 min at 700 × *g* and 4 °C, and the supernatant was filtered through a 0.22 μm sterile filter. DNase I (0.5 U mL^−1^ final concentration, Fermentas, Vilnius, Lithuania) was added to the filtrate, which was incubated in the dark for 30 min at room temperature [4]. Subsequently, DNase was inactivated according to the manufacturer's instructions. The number of viral particles in the filtrate (Table 1 and Fig. 1E) was determined by epifluorescence microscopy after SYBR Green I staining.
6. *Viral reconcentration and deionization*. Previous studies indicated that ≥10^9^ mL^−1^ of viral particles was required for exploring the diversity of marine viruses based on metagenomic methods [5–7]. In addition, it is generally known that the extra salt ions largely affect the downstream viral enumeration and molecular biological analysis, for example, viral genomic DNA extraction, PCR reaction. Accordingly, the Centricon Plus-70 centrifugal filter device (Millipore, MA, USA) was used to reconcentrate the viral concentrate obtained from the water and settled matter samples (see Graphical Abstract, viral concentrate (I) was obtained from the water fraction using TFF concentration, and viral concentrate (II) was isolated from the settled matter fraction). The Centricon Plus-70 centrifugal filter device was firstly rinsed with 0.02 μm filtered and autoclaved MilliQ water to remove the humectant according to the manufacturer's instructions. Approximately 70 mL viral concentrate was added to each sample filter cup. Samples were centrifuged for 15 min at 3500 × *g* and 4 °C using a benchtop swinging bucket rotor (Xiangyi, Hunan, China) followed by three runs of rinsing, using 60 mL of 0.02 μm filtered and autoclaved MilliQ water for each run and a sample filter cup for deionization. The salinity of the filtrate was measured by using a conductivity meter (HACH, NYC, USA) to verify whether salt ions were removed. Finally, the viral concentrates were recovered by centrifuging at 900 × *g* and 4 °C for 2 min. The number of viral particles in the concentrate (Table 1 and Fig. 1F) was determined by epifluorescence microscopy after SYBR Green I staining.

### Verification of viral particle purity and structural integrity

1. *PCR amplification analysis and DNase I treatment*. Prior to viral nucleic acid extraction, it was necessary to check whether viral concentrates were contaminated with microbial, eukaryotic cells and/or extracellular nucleic acids. The 16S rRNA and 18S rRNA gene fragments were amplified using universal primer pairs, (i) 27F(5′-AGAGTTTGATCCTGGCTCAG-3′) and 1492R (5′-GGTTACCTTGTTACGACTT-3′) (specific for bacteria [8]), (ii) 340F (5′-CCCT AYGGGGYGCASCAG-3′) and 1000R (5′-GGCCATGCACYWCYTCTC-3′) (specific for archaea [9]) and (iii) 18SF (5′-CCGCAGCTAGGAATAATGGAATAGGAC-3′) and 18SR (5′-GTTAGCATGCCAGAGTCTCGTTCGT-3′) (specific for eukaryotes [10]). PCR amplification was performed in a total volume of 25 μL reactant containing 12.5 μL 2× PCR MasterMix (TIANGEN, Beijing, China), 0.4 μM of each primer, 9.5 μL ddH_2_O, and 1 μL of final viral concentrate as template. PCR programs were listed as follows: (i) For amplifying bacterial 16S rRNA gene fragment – initial denaturation at 95 °C for 4 min, followed by 35 cycles of denaturation at 94 °C for 45 s, annealing at 55 °C for 45 s, extension at 72 °C for 1 min, and a final extension at 72 °C for 10 min; (ii) For amplifying archaeal 16S rRNA gene fragment – initial denaturation at 98 °C for 2 min, followed by 35 cycles of denaturation at 95 °C for 30 s, annealing at 57 °C for 30 s, extension at 72 °C for 90 s, and a final extension at 72 °C for 7 min; (iii) For amplifying eukaryotic 18S rRNA gene fragment – initial denaturation at 94 °C for 5 min, followed by 35 cycles of denaturation at 94 °C for 1 min, annealing at 55 °C for 1 min, extension at 72 °C for 1 min, and a final extension at 72 °C for 10 min. Subsequently, 5 μL of each PCR product was electrophoresed at 120 V for 30 min in 1.5% (w/v) agarose gel and 1× Tris-acetate-EDTA (TAE) buffer. PCR products were visualized and photographed by using a GEL imaging system (Bio-Rad Laboraties, CA, USA).

**Fig. 2:**
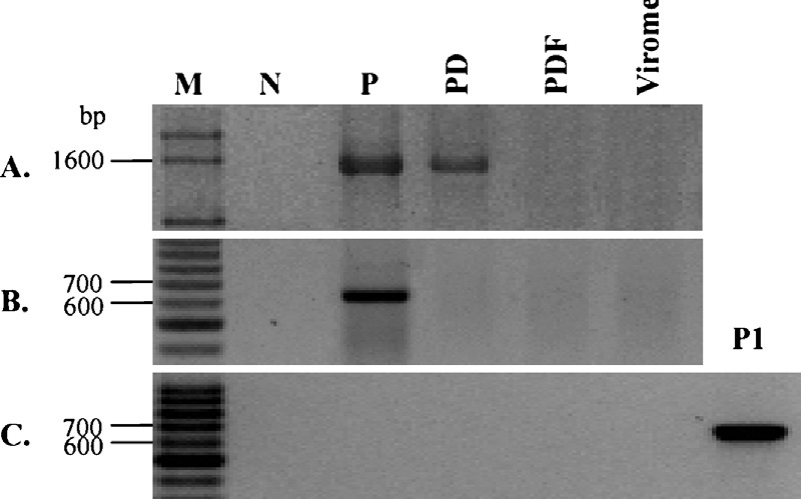
Agarose gel electrophoresis images of PCR-amplified bacterial 16S rRNA gene (A), eukaryotic 18S rRNA gene (B), and archaeal 16S rRNA gene (C) fragments. Abbreviations are as follows: M, DNA marker; N, negative control; P, final viral concentrate reconcentrated by using Centricon Plus-70 centrifugal filter device; PD, sample “P” was treated by DNase I; PDF, sample “PD” was filtered by 0.22 μm cut-off filter; P1, positive control.

The results indicated the existence of eukaryotic, microbial cells, and/or extracellular DNA contaminations (Fig. 2). To remove any potential cell (eukaryotic and microbial) contaminations introduced into the viral concentrate during the experiments as well as extracellular DNA from the lysed cells, the final viral concentrate was treated using a 0.22 μm cut-off filter and DNase I (4 U mL^−1^ final concentration). As a consequence, no obvious amplicons were observed for both 16S and 18S rRNA genes (Fig. 2).

2. *Examination of viral morphotypes.* Viral morphotypes were observed by using transmission electron microscopy. The negative staining of viral particles referred to the methods described in [11–13] with modifications. A drop (10 μL) of the viral concentrate was placed on a sheet of parafilm. A copper grid was floated on the drop for 15 min. The grid was then removed, and its edge was blotted with a piece of clean filter paper. Subsequently, the grid was stained with 2% phosphotungstic acid in 60 mM Sørensen phosphate buffer (pH 6.5) for 2 min. Excess phosphotungstic acid was removed as described above followed by air-drying for a few minutes. The grids were examined under a Philips TECNAI 12 transmission electron microscope at an acceleration voltage of 100 kV.

**Fig. 3:**
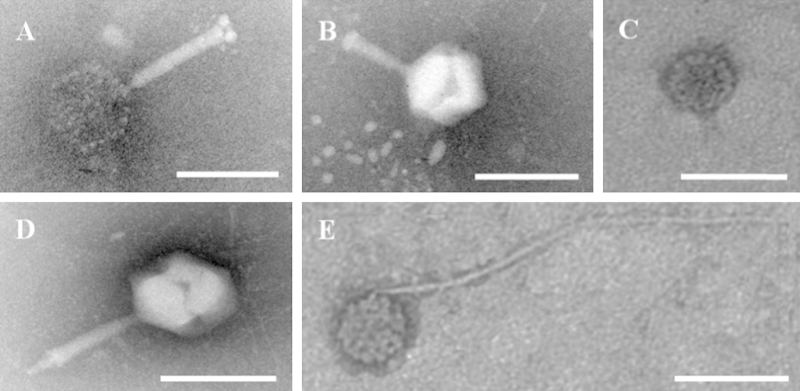
Transmission electron micrographs of phosphotungstic acid stained viruses isolated from the high turbidity seawater. (A, B and D) Myoviruses; (C) Podoviruses; (E) Siphoviruses. Scale bar = 100 nm.

Most of the observed viral particles had a distinct head-and-tail morphology (Fig. 3), which are typical features of bacterial DNA viruses. The results of transmission electron microscopy revealed structural integrity of the purified and concentrated viral particles.

### Verification of virome purity

1. *Viral genomic DNA extraction.* The viral genomic DNA was extracted from the final viral concentrate by using a DNeasy Blood & Tissue Kit (QIAGEN, Dusseldorf, Germany) according to ‘Nonnucleated’ procedure of ‘Protocol: Purification of Total DNA from Animal Blood or Cells (Spin-Column Protocol)’ with modifications. In brief, 20 μl proteinase K was firstly added to 200 μl of the final viral concentrate, and the following procedures were the same as described in the kit.
2. *PCR amplification.* The 16S and 18S rRNA genes were amplified using the obtained viral genomic DNA as the template by using above identical protocols. On agarose gel there were no obvious amplicons detected for both 16S (Fig. 2A and C) and 18S rRNA genes (Fig. 2B), which indicated that the extracted viral DNA was basically free DNA contamination of cellular organisms, and subsequently confirmed the purity of the final viral concentrate.

### Analysis of viral recovery efficiency

The viral recovery efficiency was calculated as the percentage of the number of viruses in final viral concentrate divided by the number of viruses in the original sample, which was determined with triplicate counts.

### Efficiency of the optimized method

Approximately 1 L viral concentrate (viral concentrate (I), see Graphical Abstract) was obtained from the water fraction after TFF concentration in less than 20 h. The number of concentrated viral particles was 3.11 × 10^8^ VLPs mL^−1^ (Table 1 and Fig. 1C).

For the membrane rinsing process, approximately 1 L eluant was obtained after washing with 20 L TFF permeate, and the number of viral particles was 5.98 × 10^7^ VLPs mL^−1^ (Table 1 and Fig. 1D). This part of the eluant, containing viruses trapped on the filter membrane during the first TFF, was incorporated into the viral concentrate (I).

A total of 509.8 g settled matter was separated from 150 L seawater, subjected to viral isolation (see Graphical Abstract), and contained 7.51 × 10^8^ VLPs g^−1^ (Table 1 and Fig. 1E). The total number of viral particles isolated from the settled matter (3.83 × 10^11^ VLPs, see Table 1) was slightly higher than that recovered from water fraction (3.11 × 10^11^ VLPs, see Table 1). Obviously, in order to isolate the most viral particles from high turbidity seawater, the flocculation of viral particles to suspended matter needs to be considered seriously and cannot be ignored.

To reduce the volume of viral concentrate and obtain a high density of viral particles, the whole viral concentrates were reconcentrated by using a Centricon Plus-70 centrifugal filter device. In the end, approximately 4.28 mL viral concentrate (16.30 × 10^10^ VLPs mL^−1^) (Table 1 and Fig. 1F) was obtained from 2.5 L viral concentrates (1 L from TFF concentration of the water fraction, 1 L from membrane rinsing, and 500 mL from the settled matter). After washing, the salt ions were removed from the viral concentrates. Compared to the original seawater samples, the viral recovery efficiency was 67.10%, and the concentration factor were >3.5 × 10^4^ (150 L divided by 4.28 mL) regarding water volume and 2.3 × 10^4^ (16.30 × 10^10^ VLPs mL^−1^ divided by 6.95 × 10^6^ VLPs mL^−1^) in consideration of viral density.

### Comparison of the optimized and standard methods

For the standard TFF concentration method, 150 L of water samples were successively prefiltered by gravity through eight layers of gauze and 100, 50, 25, and 10 μm pore-sized nylon fiber filters [14], and the filtrates were stored in sterile containers at 4 °C for subsequent virus concentration. The settled matter was discarded, and the rinsing process for the TFF filter membrane was not applied. Other steps involved in the concentration and reconcentration of viruses were the same as those for “water fraction” (see Graphical Abstract).

As a result, compared to the standard TFF method with a viral recovery efficiency of 30.04% (Table 1), the recovery efficiency of the optimized concentration method (67.10%) (Table 1) increased by over two times. This suggests that the method developed in this study is much more efficient in terms of purification and concentration of viruses from large body of high turbidity seawater.

### Summary

In this study, the optimized method for viral concentration from high turbidity seawater is characterized by high viral recovery efficiency (67.10%), high concentration factor (>3.50 × 10^4^), high viral particle densities (>10^11^ VLPs mL^−1^), high throughput (150 L seawater per 36 h), good structural integrity of viral particles, and the lack of other contaminants, for example particles and cells larger than 0.22 μm, extracellular nucleic acids, organic materials, and inorganic ions. The isolated viruses can be used directly for subsequent analysis, for example transmission electron microscopy observation and metagenomic sequencing. Accordingly, this optimized viral concentration method paves the way for routine investigation of viruses in a large body of highly turbid seawater and other water bodies.

## Additional information

### Background

Viruses represent the most abundant and dynamic biological components in the global ecosystems, including the marine environments [15,16]. Since viruses lack a universally conserved gene, for example the ribosomal RNA genes in cellular organisms (such as 16S rRNA gene of prokaryotes and 18S rRNA gene of eukaryotes), and since most viral hosts are nonculturable [17], traditional molecular technologies, such as denaturing gradient gel electrophoresis and clone library, are not suitable to the study of viral diversity, which make it even harder to understand marine viral diversity. To surmount these difficulties, recently, the viral particles have been isolated from seawater followed by diversity analysis with pulsed-field gel electrophoresis (PFGE) [18–20], random amplified polymorphic DNA (RAPD) technique [21] or metagenomic approaches [6,22,23]. Viral metagenomics requires high-density (viral particle densities of ≥10^9^ mL^−1^) and large-scale concentrations of seawater to obtain enough genetic material for sequencing [5,6]. An efficient and reliable method for isolation and concentration of marine viruses from seawater samples is thus particularly important for downstream molecular biological analysis.

Currently, there are dozens of approaches to viral isolation and concentration from water samples, which include adsorption-elution [24–26], chemical flocculation [14,27], ultracentrifugation [19,28], and ultrafiltration (such as tangential flow filtration (TFF)) [7]. However, the first three methods have certain inherent defects, including (i) selective adsorption of viruses to a solid matrix with focus on the detection of specific viruses [7,29], (ii) some elution buffers interfering with downstream viral enumeration and molecular biological analysis [30], and (iii) limited throughput and low viral concentration efficiency [14,31,32]. By contrast, TFF appears to be the most efficient technology for concentrating viruses from large volumes of water samples, especially in the marine environment.

However, for high turbidity seawater samples (*i.e.* seawater samples with highly suspended matter contents), such as coastal and estuary subsurface seawater [22,33,34], which have been studied frequently during recent years, direct collection of viruses by using TFF encounters difficulties or even causes damage to the expensive TFF system. The general solution to this problem in previous studies [7,14] has involved prefiltering raw natural seawater samples before TFF with a series of pore-size nylon fiber filters or wound polypropylene sediment filters to remove all settled matter and particles by gravity or by a pump-driven system. However, this procedure has drawbacks including time wasting (filter clogging causes low flow rate), increasing the chance of contamination, and low viral concentration efficiency because of lysis and settled matter adsorption of viruses. For this reason, we focus on TFF and centrifugal ultrafiltration technology and develop an efficient viral purification and concentration method for high turbidity seawater samples. The obtained viral concentrate is ideal for subsequent analysis by epifluorescence microscopy, transmission electron microscopy, and metagenomics.

## Figures and Tables

**Table 1:** Viral abundance and recovery efficiency after concentration of original high turbidity seawater samples.

Method	Viral abundance						Recovery efficiency^a^ (% ± SD)	Concentration factor (10^4^)
	Original sample (10^6^ VLPs mL^−1^ ± SD) [10^12^ VLPs ± SD]	0.3 and 0.2 μm filtrate (10^6^ VLPs mL^−1^ ± SD) [10^11^ VLPs ± SD]	TFF concentrate (10^8^ VLPs mL^−1^ ± SD) [10^11^ VLPs ± SD]	Membrane-rinsing concentrate (10^7^ VLPs mL^−1^ ± SD) [10^10^ VLPs ± SD]	Sediment (10^8^ VLPs g^−1^ ± SD) [10^11^ VLPs ± SD]	Plus-70 reconcentrate (10^10^ VLPs mL^−1^ ± SD) [10^11^ VLPs ± SD]		
Standard TFF	(6.95 ± 0.46) [1.04 ± 0.07]	(4.08 ± 0.34) [6.12 ± 0.50]	(3.82 ± 0.21) [3.82 ± 0.21]			(7.81 ± 0.78) [3.12 ± 0.31]	30.04 ± 3.19	3.75
This study		(3.19 ± 0.12) [4.79 ± 0.18]	(3.11 ± 0.28) [3.11 ± 0.28]	(5.98 ± 0.54) [5.98 ± 0.54]	(7.51 ± 0.39) [3.83 ± 0.20]	(16.30 ± 1.05) [6.98 ± 0.45]	67.10 ± 5.49	3.50

The mean values and standard deviations were calculated based on triplicate counts.

a The final viral abundance compared to the viral abundance in original samples.
